# Sepsis, Phages, and COVID-19

**DOI:** 10.3390/pathogens9100844

**Published:** 2020-10-15

**Authors:** Andrzej Górski, Jan Borysowski, Ryszard Międzybrodzki

**Affiliations:** 1 Bacteriophage Laboratory, Hirszfeld Institute of Immunology and Experimental Therapy, Polish Academy of Sciences (HIIET PAS), 53-114 Wrocław, Poland; agorski@ikp.pl (A.G.); ryszard.miedzybrodzki@hirszfeld.pl (R.M.); 2 Phage Therapy Unit, Hirszfeld Institute of Immunology and Experimental Therapy, Polish Academy of Sciences (HIIET PAS), 53-114 Wrocław, Poland; 3 Department of Clinical Immunology, Infant Jesus Clinical Hospital, 02-006 Warsaw, Poland; 4 Department of Clinical Immunology, Transplantation Institute, Medical University of Warsaw, 02-006 Warsaw, Poland

**Keywords:** sepsis, phage, COVID-19

## Abstract

Phage therapy has emerged as a potential novel treatment of sepsis for which no decisive progress has been achieved thus far. Obviously, phages can help eradicate local bacterial infection and bacteremia that may occur in a syndrome. For example, phages may be helpful in correcting excessive inflammatory responses and aberrant immunity that occur in sepsis. Data from animal studies strongly suggest that phages may indeed be an efficient means of therapy for experimentally induced sepsis. In recent years, a number of reports have appeared describing the successful treatment of patients with sepsis. Moreover, novel data on the anti-viral potential of phages may be interpreted as suggesting that phages could be used as an adjunct therapy in severe COVID-19. Thus, clinical trials assessing the value of phage therapy in sepsis, including viral sepsis, are urgently needed.

The World Health Organization (WHO) considers sepsis to be a global health priority. Recently, it has been emphasized that, despite some success in preclinical studies on experimental sepsis, no significant progress has been achieved in clinical therapy [1]. Although more than a hundred clinical trials have focused on sepsis, none of them have provided data that could be used for improvements or a cure [2]. Furthermore, the most recent data indicate that, despite best efforts to provide protocol-based care pathways, mortality from sepsis may reach nearly 40% [3].

Gaidelyte et al. showed that most of the sepsis-causing bacteria carry functional phages that are released and circulate in the blood of septicemic patients [4]. Those phages can lyse other isolates of the same bacterial strain but not the pathogenic strain in sepsis. Thus, those prophages play a role in clonal selection of pathogens in this disorder. At the same time, it appears reasonable that phage application may potentially be used in the treatment of sepsis based on both well-known anti-bacterial as well as non-bacterial activities of phages, especially those related to their anti-inflammatory and immunomodulating effects [2,5]. In fact, in addition to data obtained in experimental animals, there are already reports of successful phage therapy in patients with sepsis [2]. In this review, we summarize the progress in treating sepsis with phage therapy over the last three years.

## 1. Phage Therapy of Experimentally Induced Sepsis

Leshkasheli et al. demonstrated the therapeutic efficacy of phage therapy in *Galleria mallonella* larvae and in a mouse model of sepsis caused by *Acinobacter baumanii* [6]. Similar data were obtained by Wu et al., who showed that a phage effective against that pathogen can rescue lethal sepsis mice [7]. Interestingly, the effect of a single phage treatment (1 mL ip at a dose of 10^9^) was comparable to the effect of a phage cocktail containing 14 phages. In experiments performed by Wang et al., phage therapy applied concurrently with the inoculation of the pathogen rescued 100% of mice, whilst phage administration applied 1 h after inoculation with *A. baumanii* reduced survival to approx. 50% [8].

Phage therapy efficacy has also been studied in a mouse model of neonatal sepsis caused by Escherichia coli, Klebsiella pneumoniae, Haemophilus influenzae, Pseudomonas aeruginosa, Citrobacter freundii and Moraxella catarrhalis. A single intraperitoneal (ip) phage dose rescued 60–100% of mice depending on the phage dose. More detailed studies have revealed that a concentration as low as 0.01 MOI (multiplicity of infection) was effective in rescuing 80% of mice, whilst a 0.001 MOI dose rescued only 20% of mice. Interestingly, the mice could be rescued even when phage administration was delayed for 24 h after pathogen inoculation. While both a single phage and phage cocktails were effective, optimal results have been achieved with cocktails [9].

High effectiveness of phage therapy in the treatment of experimental sepsis induced by multidrug resistant *P. aeruginosa* was also confirmed by Alvi et al. [10]. Phage-treated bacteremic mice had a survival rate of almost 100%, and no viable pathogen could be detected at 96 h post inoculation. The authors estimate that, in order for therapy of sepsis to be efficient, phages should persist in the blood for at least 3–5 h. A native agarose gel electrophoresis was applied to assess phage surface associated with high blood persistence [11].

Phages have been shown to upregulate gene expression of an anti-inflammatory cytokine Il-R antagonist [12] and downregulate NF-kappa B signaling [13]. Interestingly, suppressing NF-kappa B signaling may be beneficial in an experimental mouse model of sepsis [14].

## 2. Clinical Phage Therapy of Sepsis

A 2-year-old boy with DiGeorge syndrome, recalcitrant *P. aeruginosa* bacteremia, and an allergy to antibiotics received a cocktail of two phages every 6 h intravenously (iv) for 36 h. Treatment with antibiotics, including meropenem, tobramycin, and polymyxin B, was also continued. Blood cultures turned negative and then positive again after cessation of phage therapy. The therapy was subsequently resumed, and blood cultures were reverted to negative again. Thus, in this patient, phage therapy resulted in sterilization of the blood [15].

Jennes et al. described a patient with acute kidney injury complicated by septicemia caused by colistin-only sensitive *P. aeruginosa* and acute kidney failure. The patient received a 50 mL cocktail of two phages in a 6 h iv infusion for 10 days. Blood cultures turned negative immediately, C-reactive protein (CRP) dropped, fever disappeared, and renal function recovered [16].

Recently, Australian authors have reported results of adjunctive phage therapy (combined with antibiotics) of *S. aureus* bacteremia in 13 patients, most of whom suffered from infective endocarditis. A phage cocktail composed of three phages was administered twice daily for 14 days. A 10^9^ PFU infused phage dose yielded approx. 2 × 10^5^ PFU/mL of blood, which suggests that such a dose may be adequate to achieve a desired therapeutic effect. The iv infusions were well tolerated; no fever, rashes, hypotension or other adverse reactions were observed. Clinical improvement was evident in eight of the 13 patients, and inflammation markers declined during or soon after the therapy [17].

Further progress in phage therapy of sepsis has recently been achieved by introducing engineered phages used to treat a patient with a disseminated drug resistant mycobacterial infection. Genome engineering and forward genetics were applied to obtain lytic phage derivatives that were infused iv as a three-phage cocktail (10^9^ PFU per dose of each phage) twice daily for 32 weeks. Phage infusion was well tolerated, without significant side effects. Serum phage titers reached levels exceeding 10^9^/mL. Weak anti-phage protein antibody responses were noted, but no evidence of phage neutralization was observed. The clinical condition of the patient improved while sterilization of the blood and sputum was achieved [18].

Sepsis is one of the principal causes of morbidity and mortality in neonates and young children in low and middle-income countries. Recently, scientists in Iraq have developed a cocktail of phages against pathogens implicated in neonatal sepsis in a Bagdad teaching hospital (*E. coli*, *K. pneumoniae*, *H. influenzae*, *P. aeruginosa*, *C. freundii,* and *M. catarrhalis*). The cocktail containing 29 phages showed activity against all bacterial hosts in vitro and should be further examined for its in vivo efficacy in experimental and clinical sepsis [19].

In summary, in animal studies, phages were administered ip at a dose of 10^9^–10^10^, and the outcome was assessed as animal rescue and reduction of bacterial burden in organs or blood. In human studies, the outcome was assessed as a clinical improvement combined with laboratory signs (e.g., disappearance of fever, improvement in general patient well-being, improvement in organ function (e.g., renal function), drop of CRP, negative blood cultures). No significant adverse effects were observed in treated patients, which is in line with our data on large cohorts of patients [20]. Phage treatments elicit antibody responses; however, those serum antibodies do not appear to significantly influence the outcome of therapy [21]. On the other hand, it cannot be excluded that phage therapy also induces the formation of a phage–antibody immune complex. Therefore, the clinical significance of a humoral response to phages during the therapy requires further studies. Furthermore, more detailed studies on phage pharmacokinetics would be helpful for the advancement of phage therapy.

Recent data suggest that disturbances in the microbiome can enhance susceptibility to sepsis [1]. Interestingly, fecal microbiota transplantation (FMT) may rescue mice from human pathogen-mediated sepsis [22]. Additionally, FMT improves survival in sepsis induced in rats [23]. Notably, it has been suggested that transfer of phages may play a role in the efficacy of FMT [24,25]. Interestingly, a clinical trial of FMT for patients with COVID-19 is ongoing [26]. Moreover, promising results have been achieved in subgroups of septic patients treated with the Il-R antagonist (28 day mortality 34.6% vs. 64.7% placebo) [1]. In addition to the data presented earlier [2], these findings seem to strengthen arguments for the potential application of phages in the treatment of sepsis.

In addition to phages, phage-derived lytic enzymes (lysins) have also been studied as a potential weapon against multi-drug resistant bacteria. Recently, the results of a first placebo-controlled clinical trial involving an antistaphylococcal lysin (exebacase) administered in conjunction with antibiotics in patients with *S. aureus* septicemia (most with infectious endocarditis) have been published. In comparison to the control (antibiotics only), the exebacase group showed higher responder rates as well as a reduction in length of stay and readmission rates. No hypersensitivity reactions to exebacase were reported. Although preexisting anti-lysin antibodies were detectable in some patients, this did not affect the efficacy and the safety of treatment. These results offer the first tangible opportunity to improve clinical results and reduce mortality in staphylococcal sepsis using phage-derived lysin [27]. Table 1 summarizes the recent progress in successfully applying phages and lysin in experimental and clinical sepsis.

## 3. COVID-19, Viral Sepsis, and Phages

Severe acute respiratory syndrome coronavirus (SARS-CoV-2) probably originated from a virus that has been circulating in horseshoe bats for several decades. It was first detected in Wuhan, China, which suggests the existence of an intermediary (pangolin?) that facilitated transmission to humans. There were also hints that the virus escaped or was deliberately released from a local institute [28]. As of 30 September 2020 there have been almost 34 million COVID-19 cases worldwide with >1 million deaths, and >25 million patients have recovered [29]. It has been noted that severe COVID-19 patients may develop typical manifestations of septic shock with blood and respiratory tract cultures testing negative for bacteria and fungus. Therefore, viral sepsis could be responsible for clinical manifestations in those patients in whom systemic cytokine storm, lymphopenia, and thrombotic complications are usually detectable. Effective antiviral therapy combined with attempts to modulate the innate immune response and upgrade the adaptive immune response are recommended to improve the outcome [30,31,32]. In fact, antiviral and anti-inflammatory treatments may be effective early in the disease [33,34].

The lungs—the primary target organ of the SARS-Cov-2 virus—are relatively accessible to phages delivered by different routes, including oral administration. However, nasal or tracheal delivery are preferred to achieve sufficient in situ concentrations. Aerosol phage preparations as well as nebulized preparations may serve as the most efficient therapeutic applications [35].

Growing data suggests that phages may interfere with the pathogenic action of eukaryotic viruses [36]. This historical data has been supported by new findings indicating a protective action of the T4 phage on human lung epithelial cells infected with human adenovirus (Adv); furthermore, adsorption of Adv to human lung and kidney epithelial cells as well as viral replication was also inhibited [37]. New data suggest that cell layers of the body may be the major sink for administered phages; interestingly, lung epithelial cells show the highest accumulation of phages [38]. Moreover, the expression of Adv genes and synthesis of Adv DNA may also be downregulated by T4 and staphylococcal phages [39]. We hypothesized that phage therapy might be helpful in combatting COVID-19 [40]. Thus, it is known that CoV-expressing cells display markedly upregulated levels of reactive oxygen species (ROS), and high levels of ROS are observed in the lungs of patients with COVID-19 [41,42]. Phages downregulate ROS production induced by bacteria and endotoxins [20]. Lymphocytopenia is frequently found in COVID-19, while the virus is known to induce apoptosis [41,43]. Moreover, autopsies have revealed atrophy of the spleen and the lymph nodes [44]. Interestingly, phages may reduce apoptosis of human airway epithelial cells when cultured in vitro [45]. In addition, Sweere et al. demonstrated that Pf phages cause upregulation of interferon (IFN) alpha and Il-12, thus promoting an antiviral signature in the lungs of mice [46]; Gogokhia et al. found that phages of *Lactobacillus, E. coli and Bacteroides* stimulate production of another potent antiviral cytokine, IFN gamma [47].

Recently, we showed that the T4 phage induces upregulation of the human defensin 2 gene (hBD2) [48]. This peptide, which is exposed primarily by epithelial cells, reduces viral replication and may enhance pathways responsible for other anti-microbial effects, both anti-bacterial and anti-viral. hBD2 activates primary anti-viral innate immune responses [49]. It suppresses HIV infection of HeLa cells in vitro [50], drastically reduces human respiratory syncytial virus (HRSV) infection of human lung epithelial cells [51], and inhibits the infectivity of HIV virions of human tonsil epithelial cells [52]. Thus, defensins have been shown to participate in antimicrobial defenses in the human respiratory tract, and the up-regulation of hBD2 may enhance those defenses [53]. Therefore, T4-induced hBD2 could also be engaged in mediating anti-SARS-CoV-2 defenses, which requires experimental confirmation.

An inflammatory response causes activation of the hemostatic system (endothelial and platelet activation and coagulation promoting thrombosis)—a syndrome also referred to as thromboinflammation which is relevant in COVID-19 [54]. In fact, thrombocytopenia is associated with increased risk of severe disease [55]. Platelets are known to interact with viruses and have recently been shown to be transient carriers of HIV, thus contributing to HIV dissemination by propagating the virus to macrophages. This process could be prevented by the anti-integrin alphaIIb/beta3 antibody [56]. Coronaviruses may also infect bone marrow cells [55]. SARS-CoV RNA may be present in platelets of COVID-19 patients, which suggests that platelets can participate in the dissemination of the virus [57]. It would be of interest to determine if this phenomenon could also be blocked by the anti-alphaIIb/beta3 antibody, which has been effective in preventing HIV dissemination. The alphaIIb/beta3 integrin binds specifically to a KGD (Lys-Gly-Asp) sequence motif exposed on the gp24 capsid protein of T4 phages. Therefore, T4 phages could interfere with platelet-dependent SARS-CoV dissemination, mimicking the effect of the anti-alphaIIb/beta3 antibody. In fact, it has been demonstrated that such interference could enable T4 phages to reduce the adhesion of platelets to fibrinogen [13].

Interestingly, there appears to be another target for anti-COVID-19 effects of phages. SARS-CoV-2 binds to its receptor, angiotensin-converting enzyme 2 (ACE2), through the receptor-binding domain (RBD) present in its major structural protein spike. Recently, an exposed KGD motif has been identified in ACE2, thus enabling it to interact with integrin alphaIIb/beta3 [58]. Therefore, platelets could associate with the SARS-CoV-2–ACE2 complex using their alphaIIb/beta3 integrin receptor targeting the KGD sequence present within the ACE2 molecule. This phenomenon could contribute to SARS-CoV-2 dissemination and upregulate platelet-mediated coagulopathy and tissue injury. The presence of platelet-fibrin thrombi is common in lung lesions in patients with COVID-19 and is considered to be the main target of therapy [59]. Occupation of the platelet alphaIIb/beta 3 integrin receptor by the phage KGD could inhibit platelet engagement with the complex formed by the virus and its receptor and prevent the ensuing pathology.

## 4. Conclusions

In recent years, a number of reports derived from experimental studies in animals and human clinics have suggested the potential value of phage therapy in the treatment of sepsis. The activity of both anti-bacterial and non-bacterial phages is relevant for successful phage therapy. The anti-inflammatory and the immunomodulating properties of phages could also be useful in the treatment of severe COVID-19 syndrome including viral sepsis (Table 2). A recent article from China concludes that phage therapy in sepsis treatment can be expected in the near future [60]. The data discussed in this review support this assumption. As pointed out, relevant clinical trials assessing the therapeutic value of phage therapy in those clinical settings are urgently needed [40].

## Figures and Tables

**Table 1:** Summary of recent data on successful application of phages and lysin in experimental and clinical sepsis. ip—intraperitoneal application; iv—intravenous application.

Animal Model/Human Disease	Pathogen	Route and Schedule of Phage Administration	Reference
Galleria mellonella larvae mouse	*Acinetobacter baumannii*	single injection single ip MOI of 100	[6]
mouse	*Acinetobacter baumannii*	single ip 10^9^	[7]
mouse	*Acinetobacter baumannii*	single ip MOI of 0.1–10	[8]
mouse	*Escherichia coli, Klebsiella pneumoniae, Haemophilus influenzae, Pseudomonas aeruginosa, Citrobacter freundii, Moraxella catarrhalis*	single ip MOI of 0.001–2	[9]
mouse	*Pseudomonas aeruginosa*	single ip 10^9^	[10]
Di George syndrome with septicemia	*Pseudomonas aeruginosa*	iv for 3 days 3 × 10^5^ every 6 h	[15]
Acute kidney failure with septicemia	*Pseudomonas aeruginosa*	iv for 10 days concentration not given	[16]
Endocarditis with septicemia (13 patients)	*Staphylococcus aureus*	iv for 14 days	[17]
Disseminated mycobacterial infection	*Mycobacterium abscessus*	iv for 32 weeks 10^9^ every 12 h for 32 weeks	[18]
Endocarditis with septicemia (119 patients)	*Staphylococcus aureus*	Anti-staph lysin iv 0.12–0.25 mg/kg for 14 days	[27]

**Table 2:** The relevant targets by which phage therapy may be applicable for treatment of COVID-19.

1. Phages can interact with epithelial cells and protect those cells from virus-induced damage and apoptosis; this could be especially relevant for lung epithelial cells.
2. Phages may prevent viral adsorption to epithelial cells and downregulate viral replication in those cells.
3. Phages may induce production of cellular chaperones protecting cells from viral injury (e.g., induction of Hsp70 in human alveolar cells).
4. Phages inhibit inflammation (downregulation of Nuclear Factor (NF) kappa B and reactive oxygen species (ROS) production).
5. Phages induce anti-viral immunity (e.g., induction of interferon (IFN)-alpha and IFN-gamma, defensin 2 and inhibition of Hsp90).
6. Phages may interfere with severe acute respiratory syndrome coronavirus (SARS-CoV)-b2 binding to angiotensin-converting enzyme 2 (ACE2).
